# Thylakoid Targeting Improves Stability of a Cytochrome P450 in the Cyanobacterium *Synechocystis* sp. PCC 6803

**DOI:** 10.1021/acssynbio.4c00800

**Published:** 2025-03-02

**Authors:** Sayali S. Hanamghar, Silas Busck Mellor, Lisbeth Mikkelsen, Christoph Crocoll, Mohammed Saddik Motawie, David A. Russo, Poul Erik Jensen, Julie A. Z. Zedler

**Affiliations:** † Synthetic Biology of Photosynthetic Organisms, Matthias Schleiden Institute for Genetics, Bioinformatics and Molecular Botany, Friedrich Schiller University Jena, 07743 Jena, Germany; ‡ Department of Plant and Environmental Sciences, University of Copenhagen, 1871 Frederiksberg, Denmark; § Bioorganic Analytics, Institute for Inorganic and Analytical Chemistry, Friedrich Schiller University Jena, 07743 Jena, Germany; ∥ Department of Food Science, University of Copenhagen, 1958 Frederiksberg, Denmark

**Keywords:** cyanobacteria, cytochrome P450, Synechocystis sp. PCC 6803, PetC1, *p*-hydroxyphenylacetaldoxime, CYP79A1, Rieske protein, light-driven catalysis

## Abstract

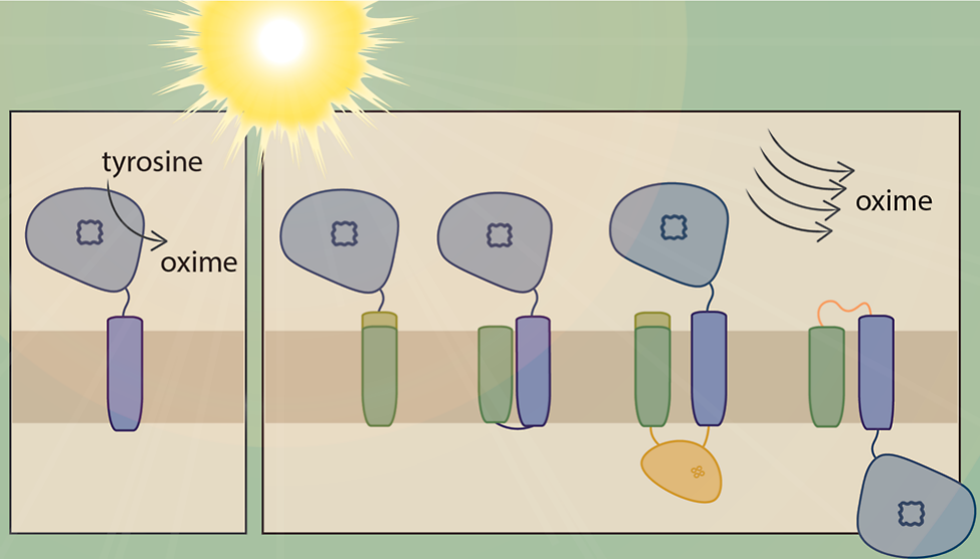

Plants produce a large array of natural products of biotechnological interest. In many cases, these compounds are naturally produced at low titers and involve complex biosynthetic pathways, which often include cytochrome P450 enzymes. P450s are known to be difficult to express in traditional heterotrophic chassis. However, cyanobacteria have shown promise as a sustainable alternative for the heterologous expression of P450s and light-driven product biosynthesis. In this study, we explore strategies for improving plant P450 stability and membrane insertion in cyanobacteria. The widely used model cyanobacterium *Synechocystis* sp. PCC 6803 was chosen as the host, and the well-studied P450 CYP79A1 from the dhurrin pathway of *Sorghum bicolor* was chosen as the model enzyme. Combinations of the P450 fused with individual elements (e.g., signal peptide, transmembrane domain) or the full length cyanobacterial, thylakoid-localized, protein PetC1 were designed. All generated CYP79A1 variants led to oxime production. Our data show that strains producing CYP79A1 variants with elements of PetC1 improved thylakoid targeting. In addition, chlorophyll-normalized oxime levels increased, on average, up to 18 times compared to the unmodified CYP79A1. These findings offer promising strategies to improve heterologous P450 expression in cyanobacteria and can ultimately contribute to advancing light-driven biocatalysis in cyanobacterial chassis.

## Introduction

Plants are known to produce a plethora of bioactive natural products including terpenoids, alkaloids, flavonoids, and phenylpropanoids. Many of these are of commercial interest as biopesticides, pharmaceuticals, fragrances or as flavoring and coloring agents.^1−3^ The biosynthesis of these compounds often requires multienzyme cascades facilitated by efficient metabolon formation.^4^ Many of these pathways include members of the cytochrome P450 (P450) superfamily. P450s are highly abundant and diverse monooxygenases that are widespread in the plant kingdom.^5,6^ In plants, P450s are typically membrane-bound in the endoplasmic reticulum and receive electrons for activity from a NADPH-cytochrome P450 oxidoreductase (CPR).^7,8^

To biotechnologically produce such plant-derived compounds, a pathway of interest is usually transferred into a suitable host chassis. Typically, this would be an established microbial system such as *Escherichia coli* or yeast.^3,9^ However, eukaryotic P450s are known to be challenging to express in traditional heterotrophic chassis in high yields in an active form.^7,10^ One of the reasons for this is the dependence on CPR, and ultimately NAD(P)H availability, for P450 activity. To circumvent the rate-limiting dependence on NAD(P)H, it has been shown that photosynthesis-derived reducing power can be harnessed to drive P450s.^11,12^ More specifically, it has been shown that photoreduced ferredoxin serves as the reductant for P450 enzyme catalysis.^7,12−18^ One of the models for light-driven catalysis is CYP79A1, part of the dhurrin pathway found in *Sorghum bicolor*, which catalyzes the conversion of tyrosine to *p*-hydroxyphenylacetaldoxime (hereafter “oxime”).^19^ CYP79A1 has been expressed in a range of photosynthetic hosts including *Nicotiana* spp.,^12,16,20,21^ the eukaryotic microalga *Chlamydomonas reinhardtii,*(22) and model cyanobacteria.^18,23,24^ Over the past decade, light-driven catalytic systems have been developed in various hosts with additional redox enzymes beyond P450s and with an expanded range of target products.^25,26^

Cyanobacteria have gained interest as chassis for sustainable biotechnology and whole cell biocatalysis.^27−29^ This is mainly owed to their photoautotrophic growth capacity, fast growth rates, and genetic tractability of several cyanobacterial species. Cyanobacteria have also been used to successfully express various P450s,^14,18,23,24,30,31^ as well as non-P450 monooxygenases^32−36^ and reductases.^37−39^ Therefore, light-driven catalysis using cyanobacteria is a proven concept with many promising future applications.^40^ Now, to advance these systems, the development of strategies to improve the catalytic process as well as the compatibility with the host cell metabolism are required. In this study, we focused on improving the localization and stability of a model P450 of plant origin, CYP79A1, by fusing it with native cyanobacterial elements. Generally, the fusion of a gene of interest (GOI) to a highly expressed native sequence is a common strategy for improving recombinant protein production across various hosts including cyanobacteria.^41,42^ Additionally, given that CYP79A1 is membrane-bound in its native host, we reasoned that successful targeting to the thylakoid membrane would be important for protein stability and ensuring access to reducing power from the photosynthetic electron transport chain.

Here, we use the established model cyanobacterium *Synechocystis* sp. PCC 6803 (hereafter *Synechocystis*) as a host to address said targeting and stability by fusing CYP79A1 to elements of the thylakoid-localized PetC1, the major Rieske protein in the Cytochrome *b*_6_*f* complex. We show that the fusion of CYP79A1 to the full length PetC1 protein results in higher oxime levels compared with the unmodified CYP79A1 enzyme. Optimized designs utilizing only certain elements of PetC1 led to improved targeting and stability of CYP79A1 in thylakoid membranes, yielding higher steady state protein and oxime levels. Our work shows that the fusion of heterologous enzymes to native cyanobacterial elements can modulate protein levels as well as the location and activity of the target enzyme relevant for improving in vivo light-driven catalysis.

## Materials and Methods

### Strains and Growth Conditions

A glucose-tolerant, nonmotile, substrain “Kaplan” of *Synechocystis* sp. PCC 6803 was used (originally obtained from Prof. Patrik Jones, Imperial College London) for the generation of all cyanobacterial strains. Cyanobacterial cultures were grown in BG-11 medium^43^ supplemented with 5 mM 4-(2-hydroxyethyl)-1-piperazineethanesulfonic acid (HEPES), pH 7.5 (BG-11_H_ medium). For maintenance, cultures were grown on 1.5% (w/v) agar plates of BG-11_H_ medium supplemented with 3 g L^–1^ sodium thiosulfate pentahydrate. Agar plates were incubated in a growth chamber at 30 °C with continuous illumination of approximately 30–40 μmol of photons m^–2^ s^–1^. Liquid cultures for experiments were grown in BG-11_H_ medium at 30 °C in glass tubes illuminated with approximately 50 μmol of photons m^–2^ s^–1^ and bubbled with 3% (v/v) CO_2_-supplemented air if not stated otherwise. Cultures of generated cyanobacterial transformants were supplemented with 50 μg mL^–1^ spectinomycin.

All cloning was performed using *E. coli* strain NEB 5-alpha (New England Biolabs) for plasmid propagation. For conjugation, the *E. coli* strain HB101 with the helper plasmid pRL443^44^ was used. All *E. coli* strains were grown in LB Broth (Miller) with the respective antibiotics (50 μg mL^–1^ spectinomycin (strains containing expression vectors) or 100 μg mL^–1^ ampicillin (strains containing the pRL443 helper plasmid).

### Generation of Constructs

All plasmids generated in this study use the RSF1010-based shuttle vector pDF-*trc*.^45^ The codon-optimized sequence of CYP79A1 from *S. bicolor* (UniProt ID: Q43135) was amplified from the previously generated plasmid “operon”^18^ by PCR. For all PCR reactions, Phusion high-fidelity DNA polymerase (New England Biolabs) was used. The vectors pJZ94 and pJZ95 were generated by restriction digestion cloning. *Eco*RI and *Hin*dIII recognition sites were added to the insets by PCR and ligated into the digested pDF-*trc* backbone using T4 DNA Ligase (Roche). Details of primers used for PCR amplification of the insets are shown in Table S1. The vectors pJZ99, pJZ100, pJZ101, and pJZ102 were assembled using overlap extension PCR cloning.^46^ To assemble pJZ99, pJZ100, and pJZ101, a megaprimer was generated by PCR using the primers listed in Table S1 followed by an overlap PCR reaction using pJZ94, pJZ95, and pJZ100 as templates, respectively. pJZ102 was generated by a one-step overlap PCR reaction using pJZ101 as a template and the primers PTCYP6-ovF and PTCYP6-ovR (Table S1). All overlap PCR reactions were digested with DpnI prior to transformation into NEB 5-alpha cells. The sequence of all generated constructs was verified by Sanger sequencing (GATC Eurofins).

### Transformation and Colony PCR of Cyanobacteria

All expression vectors were introduced into *Synechocystis* using a standard triparental mating protocol as previously described.^47^ Regarding the *E. coli* strains, HB101 carrying the pRL443 plasmid^44^ (helper strain) and a NEB 5-alpha strain carrying the respective expression vector (cargo strain) was used. Colonies that grew on BG-11_H_ agar plates supplemented with 50 μg mL^–1^ spectinomycin were further analyzed by colony PCR.

Total DNA was extracted by boiling a cyanobacterial colony in 50 μL of TE buffer at 95 °C for 10 min. Subsequently, 2 μL of the DNA extract were used in a 25 μL PCR reaction using Phusion Hot Start Flex DNA polymerase (New England Biolabs) and the primers trc-F and trc-R following previously established protocols.^34^

### Growth and Pigment Content Analysis

Strains were grown in a Multicultivator MC1000 (Photon Systems Instruments) in glass tubes at 30 °C and bubbled with 3% CO_2_-supplemented air for 10 d. For illumination, a linear gradient from 30 to 100 μmol of photons m^–2^ s^–1^ over 72 h followed by constant illumination with 100 μmol of photons m^–2^ s^–1^ for 168 h was used. For analysis of growth, cultures were inoculated in triplicate to an optical density at 750 nm (OD_750_) of 0.3 from a liquid preculture. After 24 h of growth, cultures were induced with 0.1 mM isopropyl β-*d*-1-thiogalactopyranoside (IPTG). To monitor growth, samples were harvested every 24 h to measure the optical density (OD_750_). Chlorophyll and carotenoid content of samples were estimated using a previously described protocol^48^ with the following modifications: for all samples, an equivalent volume of OD_750_ = 0.5 cells were harvested. Samples were centrifuged at 13,000*g* for 10 min at 4 °C, and the pellet was resuspended in 100 μL of 100% prechilled methanol. Samples were incubated for 30 min on ice in the dark and centrifuged again at 13,000*g* for 10 min at 4 °C.

### Preparation of Cleared Cell Lysates, Protein Content Estimation, and SDS-PAGE

To prepare cellular lysates, a culture volume equivalent to OD_750_ = 3 was harvested by centrifugation at 4000*g* and 4 °C for 10 min. Cleared lysates were prepared as previously described^34^ with slight modifications of the Bullet Blender settings to three cycles of 5 min intervals at level 12. The protein content of samples was estimated using a Pierce bicinchoninic acid protein assay kit (Thermo Scientific) following the manufacturer’s instructions in a 96-well plate format.

### SDS-PAGE, Immunoblotting, and Densitometry

Samples for SDS-PAGE were mixed with sample loading buffer (2% SDS and 0.1 M dithiothreitol) and incubated for 10 min at room temperature. Proteins were separated by SDS-PAGE as described elsewhere.^49^ Proteins separated by SDS-PAGE were then transferred onto a 0.2 μm nitrocellulose membrane by using a Trans-Blot Turbo system (Bio-Rad Laboratories) at 25 V for 7 min. Blocking, washing, and incubation steps were performed as previously described.^49^ Depending on the protein target, the following primary antibodies were used: a custom-made CYP79A1 antibody (gift from Tomas Laursen,^50^ dilution 1:5000), anti-PsbA (Agrisera AS05 084, dilution 1:10,000), or anti-RbcL (Agrisera AS03 037, dilution 1:10,000). As secondary antibody, an anti-rabbit horseradish peroxidase-conjugated antibody (Promega) was used for all blots at a dilution of 1:5000. The HRP signal was developed by using a Clarity Max ECL chemiluminescence reagent (Bio-Rad Laboratories) and detected with a ChemiDoc imaging system (Bio-Rad). For densitometric analysis, the software Image Lab (v6.1, Bio-Rad Laboratories) was used. The protein band detected for variant 1 (CYP79A1) was set to 1 and used as a reference band for relative quantitation of protein bands detected in variants 3, 4, and 6. Relative protein quantities were then adjusted to account for different amounts of protein loaded to obtain the final protein equivalents. To estimate oxime amounts per protein abundance, measured oxime values in mg L^–1^ were divided by protein equivalents.

### Fractionation of Cellular Lysates and Thylakoid Enrichment

Cultures for fractionation were inoculated to an OD_750_ of 0.5 from a liquid preculture grown in the same conditions. The same conditions as those for growth analysis were used with the modification of constant illumination with 50 μmol of photons m^–2^ s^–1^. Cultures were harvested once they reached an OD_750_ between 2 and 3. Fractionations were performed based on previously described protocols^14,34^ with further modifications. In brief, cultures were harvested by centrifugation at 4000 rpm for 10 min at 4 °C. Cell pellets were resuspended in 10 mL of lysis buffer (20 mM Tris–HCl pH 7.5, 5% (v/v) glycerol). Glass beads (diameter 0.25–0.3 mm) were added to the resuspended cells at approximately one–fourth of the lysate volume. Cells were lysed by vortexing at a maximum speed in four cycles of 10 min with intermittent incubation on ice. Subsequently, cell debris was removed by centrifugation at 3000 rpm for 5 min at 4 °C. The supernatant containing the cellular lysate was then centrifuged at 23,700 rpm for 1 h at 4 °C using an SW 40Ti rotor in a Sorvall Discovery 90SE Ultracentrifuge. The resulting supernatant with the soluble fraction was collected in a fresh tube (soluble cytosolic fraction), and the pellet (total membrane fraction) was resuspended in lysis buffer and loaded on a two-phase sucrose gradient with 30% (w/v) and 50% (w/v) sucrose layers. The sucrose gradients were then centrifuged at 29,500 rpm and 4 °C overnight (SW 40Ti rotor, Sorvall Discovery 90SE Ultracentrifuge). Thylakoid-enriched membrane fractions were recovered from the interface of 30% and 50% sucrose layers. All fractions were further processed for SDS-PAGE and immunoblot analysis, as described above.

### Topology Assay

Thylakoid-enriched membrane fractions were digested with trypsin in the presence or absence of a membrane-solubilizing detergent. Protein content of thylakoid-enriched membrane fractions was estimated as described for cleared cellular lysates. Per assay, 20 μg of the thylakoid-enriched membrane fraction was digested with 2 μg trypsin (sequencing grade modified trypsin, Promega) with or without the addition of Triton X-100 to a final concentration of 1%. Samples were digested at 37 °C for 30 min. Sample loading buffer was directly added to stop the digestion after incubation, and samples were subjected to SDS-PAGE and immunoblotting as described above.

### LC–MS Analysis of *p*-Hydroxyphenylacetaldoxime

For analysis of the *p*-hydroxyphenylacetaldoxime content in the culture medium, *Synechocystis* cultures were harvested by centrifugation at 4000 rpm for 10 min at 4 °C on day 7 of cultivation. The supernatant was mixed with methanol (HPLC-grade) to a final concentration of 25% (v/v) methanol and stored at −80 °C before further processing. *P*-Hydroxyphenylacetaldoxime was quantified with modifications from previously described methods.^21,50^ Briefly, chromatography was performed on a 1290 Infinity II UHPLC system (Agilent Technologies). Separation was achieved on a Kinetex Biphenyl column (100 mm × 2.1 mm, 1.7 μm, 100 Å, Phenomenex, Torrance, CA, USA). Ammonium acetate (2 mM, pH 6.6) and methanol were employed as mobile phases A and B, respectively. The elution profile was 0.0–0.05 min, 5% B; 0.05–4.5 min, 5–35% B; 4.5–5.0 min 35–75% B, 5.0–5.1 min 75–98% B, 5.1–6.1 min, 98% B, 6.1–6.2 min, 98–5% B, and 6.2–7.5 min 5% B. The mobile phase flow rate was 400 μL min^–1^. The column temperature was maintained at 40 °C. The liquid chromatography was coupled to an Ultivo triple quadrupole mass spectrometer (Agilent Technologies) equipped with a Jetstream electrospray ion source (ESI) operated in positive ion mode. The instrument parameters were optimized by infusion experiments with pure standards. The ion spray voltage was set to 4000 V. Dry gas temperature was set to 325 °C, and dry gas flow was set to 13 L min^–1^. Sheath gas temperature was set to 250 °C, and sheath gas flow was set to 12 L min^–1^. Nebulizing gas was set to 60 ψ. Nitrogen was used as dry gas, nebulizing gas, and collision gas. Multiple reaction monitoring (MRM) was used to monitor analyte precursor ion → fragment ion transitions. One fragment ion was used as quantifier, and two fragment ions were used as qualifiers [*p*-hydroxyphenylacetaldoxime + H]^+^*m*/*z* 152.1→ 134 (16 eV), 152.1 → 107 (28 eV) and 152→ 77.1 (44 eV). Fragmentor voltage was set to 63 V. MRM settings were optimized by the injection of a reference standard. Both Q1 and Q3 quadrupoles were maintained at unit resolution. Mass Hunter Quantitative Analysis for QQQ software (Version 10.1, Agilent Technologies) was used for data processing. Quantification was performed using standard curves ranging from 30 to 13,000 ppb of authentic *p*-hydroxyphenylacetaldoxime, prepared as previously described.^21^

## Results

### Design of CYP79A1 Variants Using a Fusion Protein Approach for Improved Thylakoid Localization

**Figure 1:**
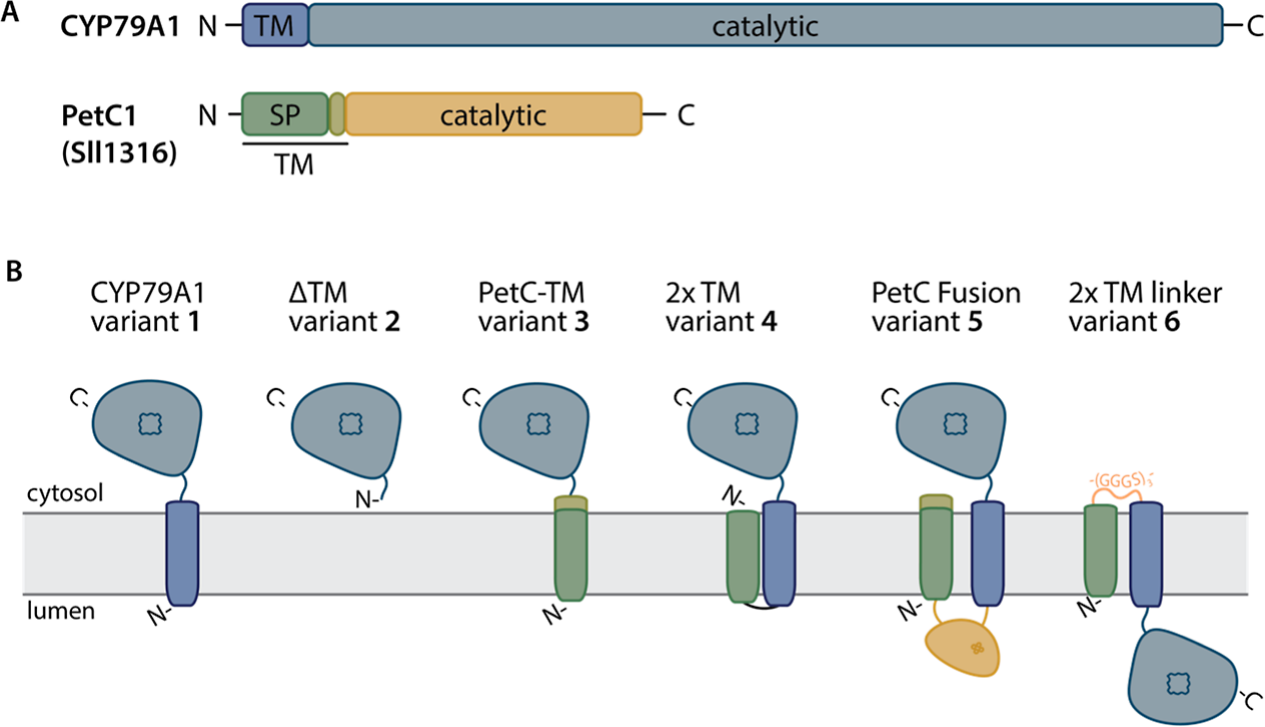
Schematic representation of CYP79A1 variants and fusions with PetC1 (Sll1316) used in this study with their predicted topology. (A) Scheme of functional domains in the unmodified sequences of CYP79A1 and PetC1 used for fusion design in this study. (B) Predicted topology of generated variants. Original sequence elements of CYP79A1 and PetC1 are shown in colors based on the elements described in panel A. The orientation of the proteins in the thylakoid membrane (shown in gray) is indicated, and the orientations of N- and C-termini are annotated. Topology predictions were done with the THMM 2.0 server^57^ (Figure S1). TM denotes a transmembrane domain, SP denotes a signal peptide, and the orange linker in variant 6 denotes a flexible (GGGS)_3_ linker.

The unmodified CYP79A1 enzyme was previously found in the thylakoid membrane fraction when expressed in cyanobacteria, but protein accumulation and activity were relatively low.^18,23^ It was speculated that improper membrane targeting of CYP79A1 could explain the low steady state protein levels. Thus, heterologous protein production might be increased if P450 is more efficiently targeted to the thylakoid membrane. We aimed at achieving this by the fusion of CYP79A1 to a native, thylakoid-localized protein. To find a suitable fusion partner, we looked at native, highly abundant proteins in the thylakoid membrane of *Synechocystis*. Candidates were further narrowed to substrates of the twin arginine translocation (Tat) machinery because Tat-dependent translocation occurs in a folded state and might include a proof-reading function.^51^ This could further aid the accumulation of active P450 holoenzyme (i.e., with the heme cofactor incorporated) in the thylakoid membrane. Based on these criteria, we chose the major Rieske iron–sulfur protein PetC1 (Sll1316) of the *Synechocystis* Cytochrome *b*_6_*f* complex. PetC1 is a member of the Rieske protein family in *Synechocystis*.^52,53^ It is an experimentally verified Tat substrate^54^ with an uncleaved signal peptide (SP) within the transmembrane domain (Figure 1A, reviewed in^55^) and a known crystal structure.^56^ We designed several different fusions between CYP79A1 and elements of PetC1 using the THMM2.0 prediction tool^57^ to predict the presence of a transmembrane domain (TM) as well as membrane topology of the protein designs (Figures 1 and S1). A schematic representation of the predicted domains and membrane orientation of all designed CYP79A1 variants is shown in Figure 1B.

The unmodified AA sequence of CYP79A1 from *S. bicolor* served as a starting point, and the encoding gene sequence was inserted into a self-replicating shuttle plasmid under the control of a *trc* promoter,^45^ as previously reported^18^ (variant 1; Table 1). Five variants were designed using the identical plasmid backbone and regulatory elements as for variant 1 modifying only the GOI sequence (Table S2). Variant 2 encoded only the soluble domain of the CYP79A1 enzyme lacking the transmembrane domain (TM) (Figure 1 and Table 1). In variant 3, the TM-encoding sequence of CYP79A1 was exchanged with the TM-encoding sequence of PetC1. For variant 4, the entire CYP79A1 sequence was kept and the predicted (uncleaved) Tat SP of PetC1^54^ was fused to the N-terminus resulting in two predicted TM segments. For variant 5, the entire PetC1 protein was directly fused to CYP79A1 with a 10×-N linker^54^ (Table 1). Finally, variant 6 was created by modifying variant 4 through the insertion of a flexible (GGGS)_3_-linker between the PetC1 and CYP79A1 elements. This insertion is predicted to alter the membrane orientation of the soluble domain, which includes the active site of the P450 enzyme (Figure 1, Table 1).

**Table 1:** Overview of Generated Variants of CYP79A1 and Fusions Thereof with PetC1 (Sll1316) for Expression in *Synechocystis* sp. PCC 6803^a^

variant #	strain name	fusion protein	predicted # of TMs^1^	predicted localization of CYP79A1 catalytic domain^b^	predicted molecular weight	expression vector
1	CYP79A1	CYP79A1 (native sequence from *Sorghum bicolor*, UniProt ID Q43135)	1	cytosol	61.9 kDa	pJZ95
2	ΔTM	CYP79A1:A2_V31del	0	n.a	58.8 kDa	pJZ94
3	PetC-TM	Sll1316:M1_P45–CYP79A1:A2_V31del	1	cytosol	63.5 kDa	pJZ99
4	2× TM	Sll1316:M1_V39–CYP79A1	2	cytosol	63.8 kDa	pJZ100
5	PetC fusion	Sll1316–10×N–CYP79A1	2	cytosol	82.1 kDa	pJZ101
6	2× TM linker	Sll1316:M1_V39–3×(GGGS)–CYP79A1	2	exterior (thylakoid lumen)	66.6 kDa	pJZ102

a Full amino acid sequences are given in Table S2.

b Based on predictions using the THMM 2.0 server.^57^

### CYP79A1 Fusion Variants are Highly Expressed in *Synechocystis*

**Figure 2:**
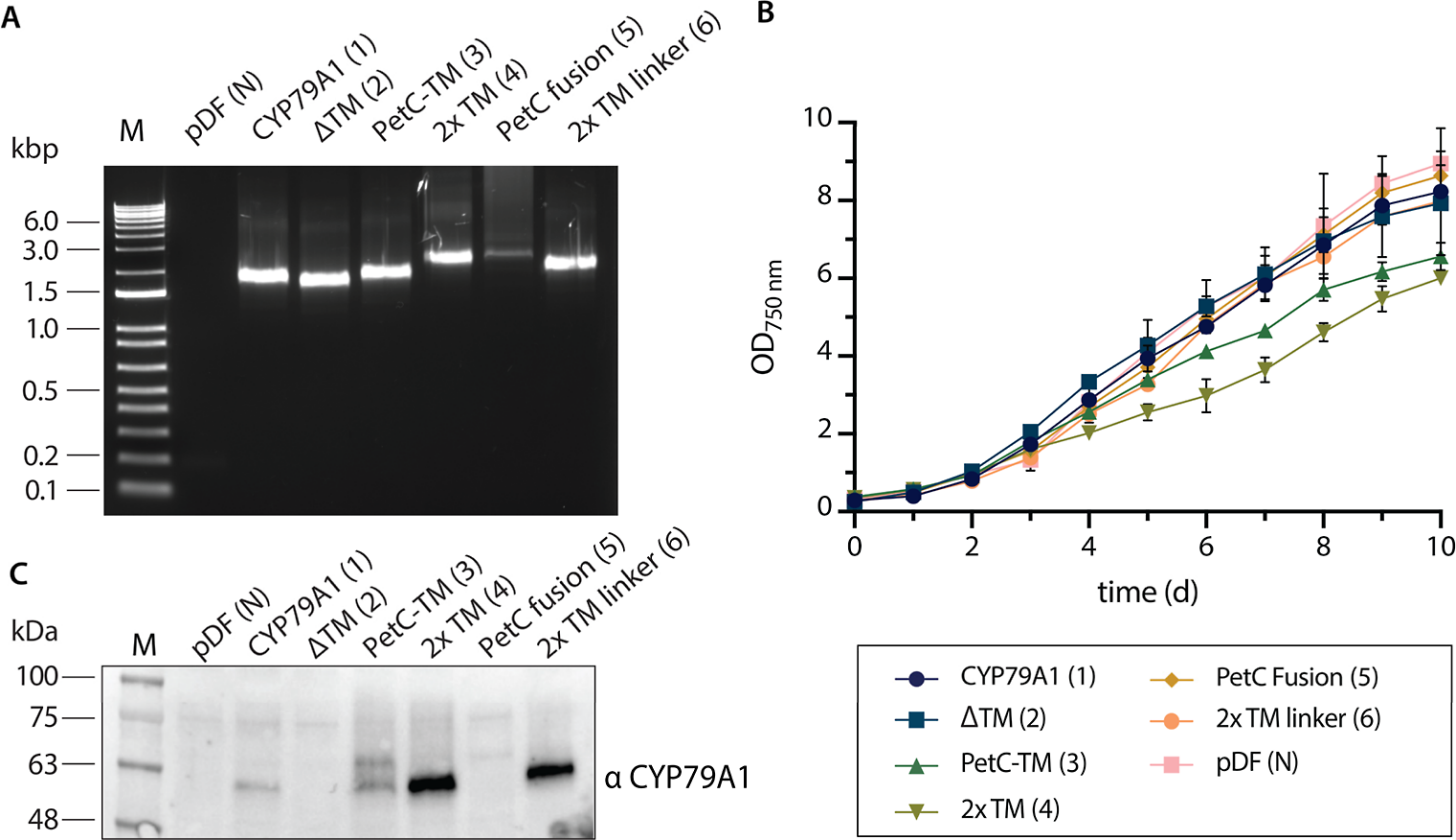
Expression of CYP79A1-PetC1 fusions in *Synechocystis* sp. PCC 6803. (A) Colony PCR of generated transformants using the plasmids pDF-trc (pDF N), pJZ95 (CYP79A1, variant 1), pJZ94 (ΔTM, variant 2), pJZ99 (PetC-TM, variant 3), pJZ100 (2× TM, variant 4), pJZ101 (PetC fusion, variant 5), and pJZ102 (2× TM linker, variant 6). (B) Growth analysis of generated *Synechocystis* strains over a period of 240 h at 30 °C under continuous illumination with 3% (v/v) CO_2_-supplemented air bubbling. Gene expression was induced with 0.1 mM IPTG after 24 h of growth. Data shown are averages from *n* = 3 biological replicates, error bars: ±SD. (C) Anti-CYP79A1 immunoblot of cleared cellular lysates harvested on day 7. In all lanes, equal amounts of 20 μg of protein were loaded except for 2× TM linker, where 10 μg were loaded.

All constructs were assembled in *E. coli,* and the generated plasmids were inserted into *Synechocystis* by conjugation to generate the respective strains termed CYP79A1 (variant 1), ΔTM (variant 2), PetC-TM (variant 3), 2× TM (variant 4), PetC fusion (variant 5), and 2× TM linker (variant 6) (Table 1). Additionally, a negative control strain carrying the plasmid backbone without an GOI, termed “pDF (N)”, was generated. The presence of the GOI sequence in the generated transformants was confirmed by colony PCR (Figure 2A).

Growth patterns of all generated strains were analyzed over a period of 10 days by monitoring the OD_750_ daily. No differences in growth compared to the negative control pDF (N) were seen in the CYP79A1, ΔTM, PetC fusion, and 2× TM linker strains (Figure 2B). However, the growth of the strains PetC-TM (3) and 2× TM (4) was slower after induction, and the final biomass yield was lower compared to the other CYP79A1 variant strains and the negative control (Figure 2B). Estimation of pigment content (chlorophyll *a* and carotenoids) on day 7 also reflected the observed growth deficit (Figure S2).

Immunoblotting with an anti-CYP79A1 antibody showed highly variable CYP7**9**A1 protein accumulation in the different strains (Figure 2C). In the CYP79A1 strain, the enzyme was detected at an expected size of approximately 62 kDa. Similar levels of CYP79A1 were observed in the PetC-TM variant. The detected CYP79A1 levels in the 2× TM variant were much higher than those found in the unmodified CYP79A1 strain. The highest expression was observed in the 2× TM linker strain. For this strain, lower amounts of lysate had to be used in immunoblot analysis to keep signal detection in a similar range (Figure 2C). Finally, although multiple colonies were tested, no CYP79A1-specific band was detectable in the ΔTM and PetC fusion strains.

Overall, all of the *Synechocystis* CYP79A1 variant strains were successfully generated, but differences in growth rates and protein accumulation were apparent depending on the specific fusion approach. Therefore, we set out to determine the impact of these differences on product levels.

### Fusion of CYP79A1 with PetC1 Elements Leads to Increased Oxime Production

**Figure 3:**
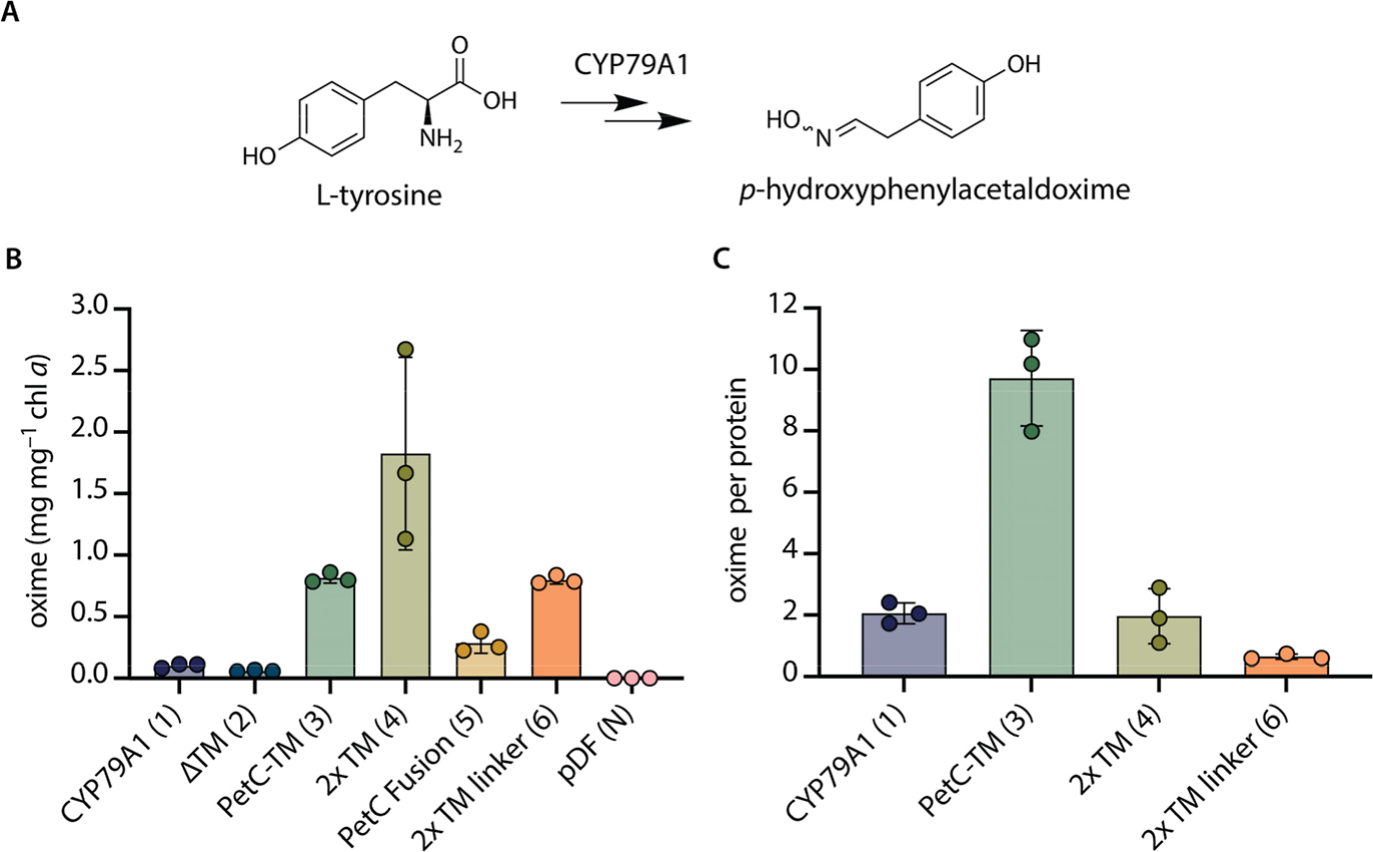
Production of (*E/Z*)-*p*-hydroxyphenylacetaldoxime (oxime) in *Synechocystis* sp. PCC 6803 CYP79A1 variant strains after 7 days of growth. (A) Overview of enzymatic reactions catalyzed by CYP79A1. (B) Oxime levels detected in cultures in mg L^–1^ normalized to chlorophyll *a* (chl *a*) content in mg L^–1^ at harvesting. (C) Oxime amounts in mg L^–1^ normalized to protein equivalents estimated by densitometry analysis (details given in Table S3) relative to unmodified CYP79A1 (variant 1) levels. Bars show averages of *n* = 3 biological replicates, error bars: ±SD with individual data points shown.

CYP79A1 sequentially converts l-tyrosine into *p*-hydroxyphenylacetaldoxime (hereafter oxime) with a total requirement of 4 electrons (Figure 3A).^19^ To confirm whether the CYP79A1 variants were active, the amount of oxime in the culture medium was quantified by LC–MS using a synthetic standard as previously described.^21^ Oxime production was detected in all strains expressing CYP79A1 fusion variants on day 7 of cultivation (Figure 3B). Altogether, chlorophyll-normalized oxime levels varied more than 30 times between CYP79A1 variants (Figure 3B).

Although the CYP79A1 protein was not detected by the antibody in strains ΔTM (2) and PetC fusion (5) (Figure 2C), oxime was detected in these strains (Figure 3B). This suggested that *CYP79A1* is expressed in both strains, but the protein accumulated below the limit of detection of the antibody used. While the absence of a TM domain (ΔTM) resulted in similar (albeit slightly lower) oxime levels, fusing CYP79A1 to the full length PetC1 protein led to almost three times higher chlorophyll-normalized oxime levels compared to the unmodified CYP79A1 strain (Figure 3B). The addition of individual elements of PetC1 in strains PetC-TM (3), 2× TM (4), and 2× TM linker (6) led to an average increase in chlorophyll-normalized oxime levels of approximately 8, 18, and 8 times, respectively. In these strains, we also observed higher protein accumulation levels (Figure 2C). Therefore, to relate protein and oxime levels, the oxime productivity was also analyzed on a per enzyme basis (Figure 3C). The increased oxime productivity found in the 2× TM (4) strain correlated with an overall higher CYP79A1 enzyme abundance, while the 2× TM linker (6) strain showed productivity levels reduced to approximately one-third of the unmodified CYP79A1 (1) (Figure 3C). The correlation of higher oxime and higher enzyme levels did not apply to the PetC-TM variant (3). In this case, an approximately 5-times higher oxime productivity per protein amount was observed (Figure 3C) despite similar CYP79A1 protein levels as the unmodified version (Figure 2C and Table S3).

### CYP79A1 Variants are Targeted to the Thylakoid Membrane

**Figure 4:**
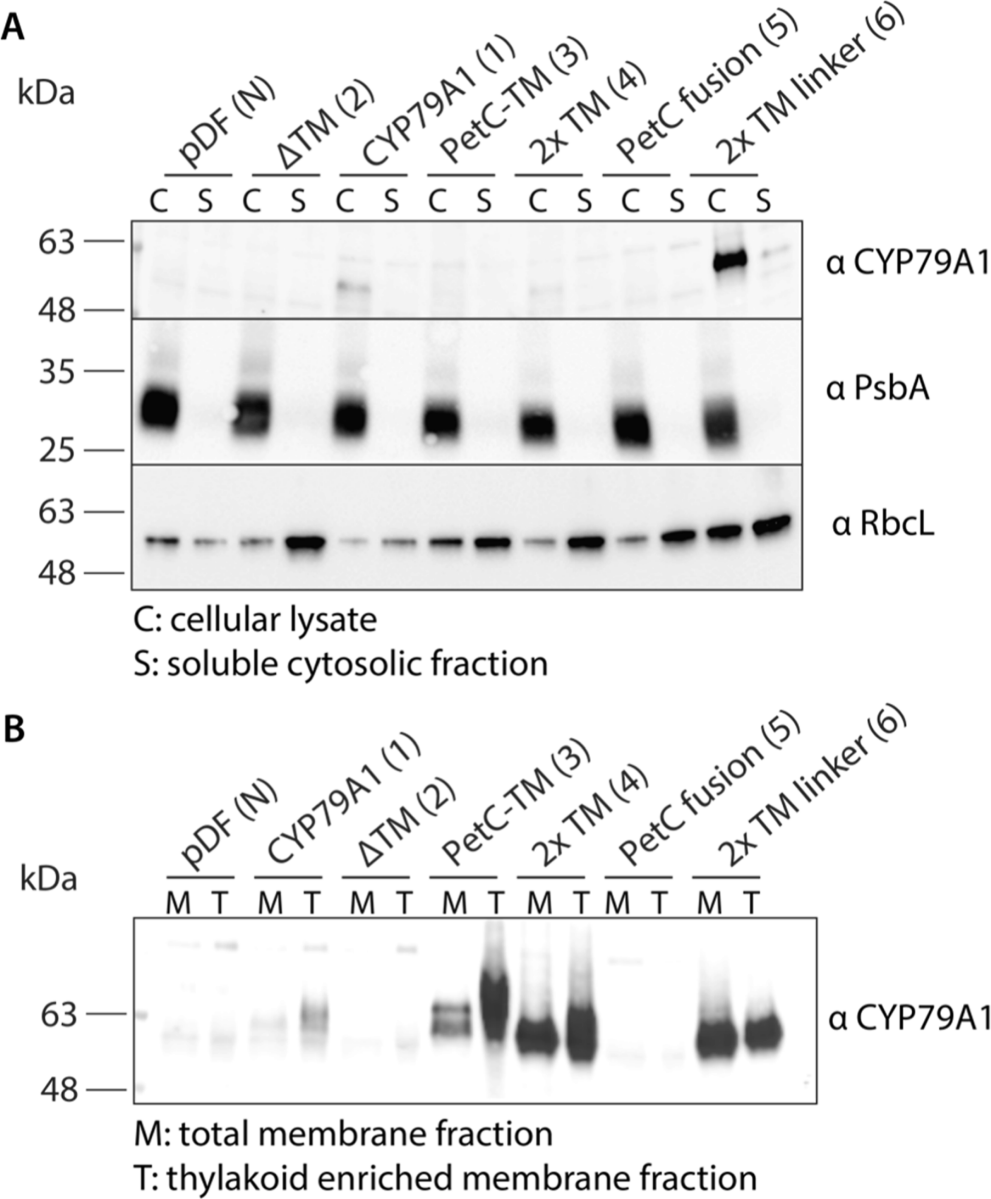
Subcellular fractionation of *Synechocystis* sp. PCC 6803 strains producing CYP79A1 variants. (A) Cellular lysate (C) and soluble cytosolic fraction (S) and (B) total membrane (M) and thylakoid enriched membrane fraction (T). For αCYP79A1 immunoblots, 20 μg of estimated protein were loaded for all samples except for panel B, where 10 μg of estimated proteins were loaded for PetC-TM (3) and 5 μg for 2× TM (4) and 2× TM linker (6). 5 μg of estimated protein were loaded for the αPsbA and αRbcL control immunoblots.

The observed differences in protein accumulation and oxime productivity across the CYP79A1 variants could be due to more efficient thylakoid membrane targeting, increased stability, and access to reducing power. Therefore, we proceeded to confirm whether the designed CYP79A1 variants were targeted to the thylakoid membrane. A subcellular fractionation protocol was adapted to first separate the soluble and membrane fractions of cellular lysates. Subsequently, the thylakoid membranes were enriched from the total membrane fraction using sucrose gradient ultracentrifugation.^14,34^ All fractions were immunoblotted with an RbcL-antibody as a marker for the soluble cytosolic fraction and a PsbA antibody as a marker for the membrane fraction (Figures 4A and S3).

The CYP79A1 variants that were previously detectable using the CYP79A1 antibody (Figure 2C) were found in the membrane fraction as expected (Figure 4B). None of the CYP79A1 variants were detected in the soluble fractions apart from a small percentage of the 2× TM linker (6) strain. This could be due to the high abundance of the protein (Figure 4A). A direct comparison of CYP79A1 abundance in total membranes compared to the enriched thylakoid fraction indicated that CYP79A1 is predominantly found in the thylakoid membranes (Figure 4B). The ratio of CYP79A1 detected in the thylakoid membranes vs total membranes was particularly high in the PetC-TM (3) strain suggesting improved targeting to the thylakoid membrane. In conclusion, the variants PetC-TM (3), 2× TM (4), and 2× TM linker (6) efficiently localized to the thylakoid membrane. However, these results are not sufficient to exclude that a small fraction of the enzyme was also found in the cytoplasmic membrane.

### Fusion Strategy can Alter Membrane Integration of CYP79A1

**Figure 5:**
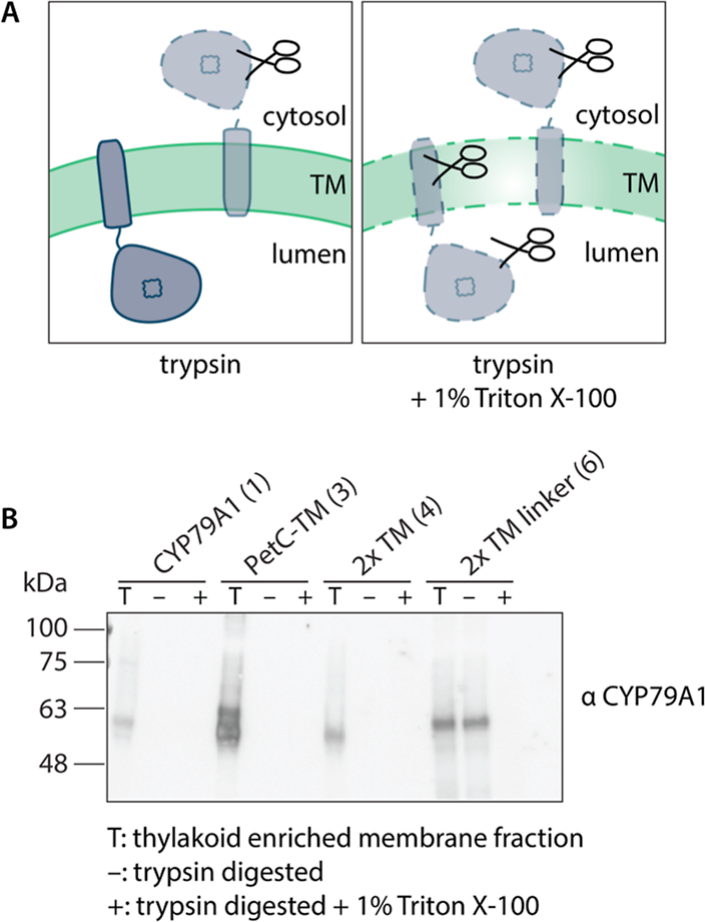
Tryptic digest assays of intact thylakoid membranes isolated from *Synechocystis* sp. PCC 6803 strains producing CYP79A1 variants. (A) Graphical representation of trypsin digestion assay for membrane topology analysis. (B) Anti-CYP79A1 immunoblot of intact isolated thylakoid membranes (T) and with trypsin digested samples in the absence (−) or presence (+) of 1% Triton X-100 for membrane solubilization.

The prediction of transmembrane domains and membrane topology (Figure S1) suggested an inverted topology of variant 2× TM linker (6) compared to the unmodified CYP79A1 enzyme (1) and the other generated variants (2–5). Additionally, a lower oxime productivity on a per enzyme basis of the 2× TM linker variant was observed (Figure 3C). We hypothesized that the activity of the CYP79A1 enzyme could be hindered if the catalytic domain was indeed localized to the thylakoid lumen, where access to reducing power and substrate availability might be altered compared to the cytosol. To investigate this, intact isolated thylakoid membranes were exposed to trypsin. The assay was performed in the presence and absence of membrane-solubilizing detergent Triton X-100. If CYP79A1 was accessible to trypsin, one would expect the degradation of the protein with and without detergent, whereas an inverted topology would protect the enzyme from tryptic digestion in the absence of the detergent (Figure 5A). We analyzed the topology of all variants that could be detected using the CYP79A1 antibody. The unmodified CYP79A1 (1) and variants PetC-TM (3) and 2× TM (4) were digested by trypsin both in the presence and absence of the detergent (Figure 5B). However, the 2× TM linker variant (6) was digested only in the presence of Triton X-100 (Figure 5B) suggesting that the catalytic domain was more protected from digestion than the other variants. This would be expected in case of an inverted topology where the catalytic domain is protected in the thylakoid lumen.

## Discussion

In this study, we aimed at improving the stability and membrane insertion of plant P450s in cyanobacteria as expression hosts. Our data showed that the fusion with native, thylakoid-targeting, elements from the Rieske protein PetC1 increased CYP79A1 enzyme levels and chlorophyll-normalized oxime productivity in *Synechocystis* sp. PCC 6803 up to 18 times. Depending on the specific fusion design, higher CYP79A1 protein levels as well as higher oxime levels were observed.

The CYP79A1-encoding gene has previously been expressed successfully in *Synechocystis* using the unmodified native amino acid sequence^18^ and as a fusion protein.^24^ In line with this, the functional expression of the plant P450 CYP79A1 was also successful in the present study. As a negative control, we generated a soluble variant ΔTM (2) that we expected to not be membrane-bound. The oxime levels in the ΔTM (2) and CYP79A1 (1) variants were similar (Figure 3B), but the ΔTM variant protein was not detected by immunoblotting (Figure 2C). Therefore, a direct correlation of the enzyme level and productivity is not possible for the ΔTM variant. It is unlikely that the lack of protein detection is derived from a lack of gene expression. The expression vector (including promoter and 5′untranslated region) was identical for all tested constructs apart from the modifications within the GOI sequence. This could be further validated using qPCR to exclude other factors altering gene expression levels derived from the GOI sequence itself. Most likely, the lack of protein detection was due to low enzyme accumulation, as the PetC-TM (3) variant had the identical AA sequence proportion from the native CYP79A1 enzyme and was detectable by immunoblotting. It is possible that the lack of a membrane anchor could decrease protein stability and increase the susceptibility of the soluble P450 to protein degradation (e.g., by native proteases). Generally, the membrane anchor in eukaryotic P450s is thought to be important for stability.^58,59^ A recent study comparing common N-terminal modification strategies for functional plant P450 expression in *E. coli* showed that the truncation of the P450 transmembrane domain led to higher oxime levels mainly due to higher protein levels.^60^ CYP79A1 was also previously expressed as a fusion construct without a TM-encoding sequence in *Nicotiana benthamiana*. However, the protein was unexpectedly found to be thylakoid membrane-associated albeit with lower protein amounts and activity levels than the unmodified variant.^21^ In *Synechocystis*, the truncation does not seem to negatively affect the enzyme activity but causes lower enzyme levels.

All variants generated by fusion with PetC1, or elements thereof, substantially increased product yields (Figure 3) and the amount of detectable protein found in the cell, apart from the PetC fusion variant (Figures 2C and 4). Interestingly, we did observe different patterns depending on the specific fusion strategy applied. Fusion with a full-length native protein is a common strategy in improving heterologous protein production, for example, fusing native cyanobacterial elements to heterologous proteins was found to substantially increase heterologous protein amounts in *Synechocystis* for plant-derived enzymes^61^ and other eukaryotic proteins.^62,63^ Therefore, a full-length PetC fusion (5) was included in the CYP79A1 variants tested in this work. Fusing CYP79A1 with PetC1 is unlikely to lead to an impairment of the native Cytochrome *b*_6_*f* complex assembly as a PetC1-GFP fusion was shown to not assemble into the Cytochrome *b*_6_*f* complex but localize to the thylakoid membrane in *Synechocystis*.^64^ This is also in line with the growth of the PetC fusion (5) strain, which is comparable to the empty vector control (Figure 2B). The PetC fusion (5) strain produced more oxime than the unmodified CYP79A1 strain. However, the lack of protein detection limits the conclusions that can be drawn. Based on the assumption that the lack of protein detection is due to low protein abundance, the PetC fusion (5) variant might have a higher activity than the unmodified enzyme.

The other fusion strategies applied in this study used only the individual elements of PetC1. The soluble domain of CYP79A1 was previously fused to the membrane anchor of photosystem I subunit PsaM in *Synechococcus* sp. PCC 7002^23^ and to elements of thylakoid-localized TatB from *Arabidopsis thaliana* for transient expression of the Dhurrin pathway in *N. benthamiana*.^20^ Both approaches were successful in producing active CYP79A1. Therefore, we tested a similar fusion approach with individual elements of the *Synechocystis* PetC1 protein (variants 3, 4, and 6). This approach led to much higher protein accumulation, between 1.2 and 21 times that of the unmodified CYP79A1 enzyme (Table S3), and increased oxime productivity (Figure 3B). Nevertheless, the specific fusion strategies led to different improvements. Exchanging the single TM of CYP79A1 (variant 3) did not have a major impact on protein levels (Figure 2C and Table S3). However, the addition of a “second” TM (variants 4 and 6) resulted in elevated CYP79A1 levels suggesting improved protein stability and/or membrane integration. Targeting to a specific membrane is challenging in cyanobacteria due to the presence of several distinct membrane systems operated by a single set of protein translocation machinery.^51,65^ Our data suggest that using elements of PetC1 improves the thylakoid membrane targeting specificity of the heterologous CYP79A1 enzyme (Figure 4).

Regarding oxime levels, PetC-TM (3) showed higher product yields regarding not only chlorophyll-normalized oxime levels but also relative values on a per enzyme basis compared to the unmodified enzyme, while in the 2× TM (4) variant, higher protein levels corresponded with higher oxime levels and the 2× TM linker (6) variant showed a slightly reduced oxime productivity on a per enzyme basis (Figure 3). Based on these nuances, we can speculate that in the PetC-TM (3) variant enzyme activity is improved due to more efficient incorporation in the thylakoid membrane along the photosynthetic electron transport chain. It has been previously shown that the removal of native competing electron sinks can improve P450 activity.^66−68^ Therefore, the P450 might better compete against native electron sinks if closely localized to the electron donor.^13^

Regarding access to reducing power, it has also been shown that different redox partners and fusions thereof with P450s can improve competitiveness with native electrons sinks and significantly enhance the catalytic activity.^24,50^ This would also be consistent with the lower oxime productivity per enzyme in the 2× TM linker (6) variant. The predicted altered membrane integration and topology (Figure 5) might negatively affect the access of the catalytically active domain to reducing power. Further experimental evidence is needed to confirm whether the membrane orientation of variant 6 is indeed inverted (Figures 1C and 4) or if a higher degree of embedding in the membrane is sufficient to explain these results.

It is also possible that the addition of elements from PetC1 improved the P450 holo-enzyme assembly. We specifically chose elements of PetC1 as this is a verified Tat substrate.^54^ Tat is important for metalloprotein biosynthesis in cyanobacteria.^69^ Therefore, targeting to the Tat pathway could aid in membrane insertion as well as proof-reading and thereby holoenzyme formation.^55^ This hypothesis will need further experimental validation in future studies.

Generally, the two key factors for successful heterologous expression of P450 enzymes are thought to be high-level functional expression and access to reducing power for catalysis.^70^ This study focused on improving the functional expression of a model plant P450 in *Synechocystis*. Going forward, it would be interesting to combine these strategies with improvement of access to reducing power. Several strategies regarding this have been applied to redox enzymes in *Synechocystis*. These include fusion of CYP79A1 to dedicated electron donors,^24^ elimination of competing electron sinks,^67^ and the evaluation of different electron carriers to fuel a P450 from *Acidovorax.*(71) Furthermore, it will be important to integrate these strategies with the host cell metabolism. Recent studies show that balancing the energy metabolism of the cell (sources and sinks) is important for generating efficient biocatalytic systems which can even improve the photosynthetic capacity of the host organism.^15,66,68,72−74^ Our findings can contribute to improving heterologous P450 expression for whole cell cyanobacterial biocatalytic systems, especially where heterologous membrane-bound enzymes driven by reducing power from the photosynthetic electron transport chain are needed.

## Data Availability

The data underlying this study are available in the published article and its Supporting Information. All other materials generated within this study are available from the corresponding author upon reasonable request.

## Supporting Information Available

CYP79A1 variant membrane topology prediction, estimation of pigment content, control blots for membrane fractionation, amino acid sequences of CYP79A1 variants, densitometry, and lists of primers used for plasmid generation (PDF)

## Abbreviations

CPR: NADPH-cytochrome P450 oxidoreductase
*E. coli*: *Escherichia coli*
IPTG: isopropyl β-*d*-1-thiogalactopyranoside
OD_750_: optical density at 750 nm
oxime: *p*-hydroxyphenylacetaldoxime
P450: cytochrome P450
*Synechocystis*: *Synechocystis* sp. PCC 6803
Tat: twin arginine translocation
