# Supplementary material for: Thylakoid Targeting Improves Stability of a Cytochrome P450 in the Cyanobacterium Synechocystis sp. PCC 6803
Source: ACS Synth Biol. 2025 Mar 2;14(3):867–77. doi: 10.1021/acssynbio.4c00800 (PMC11934225; doi:10.1021/acssynbio.4c00800)
Supplement: Supplementary file 1 — sb4c00800_si_001.pdf [file sb4c00800_si_001.pdf]

Supporting Information

Thylakoid targeting improves stability of a cytochrome P450 in the cyanobacterium *Synechocystis* sp. PCC 6803

*Sayali S. Hanamghar[1], Silas Busck Mellor[2], Lisbeth Mikkelsen[2], Christoph Crocoll[2], Mohammed Saddik Motawie[2], David A. Russo[3], Poul Erik Jensen[4] and Julie A. Z. Zedler[1]**

[1] Synthetic Biology of Photosynthetic Organisms, Matthias Schleiden Institute for Genetics, Bioinformatics and Molecular Botany, Friedrich Schiller University Jena, 07743 Jena, Germany

[2] Department of Plant and Environmental Sciences, University of Copenhagen, 1871 Frederiksberg, Denmark

[3] Bioorganic Analytics, Institute for Inorganic and Analytical Chemistry, Friedrich Schiller University Jena, 07743 Jena, Germany

[4] Department of Food Science, University of Copenhagen, 1958 Frederiksberg, Denmark.

*correspondence: julie.zedler@uni-jena.de

Overview of Content

**Figure S1**	Transmembrane helices and membrane topology prediction by the software THMM 2.0.
**Figure S2**	Estimation of pigment content.
**Figure S3**	Control blots for membrane fractionation of *Synechocystis* strains expressing CYP79A1 variants.
**Table S1**	Amino acid sequence of CYP79A1 variants used in this study.
**Table S2**	Densitometry of relative CYP79A1 protein quantities.
**Table S3**	Primers used for plasmid generation.

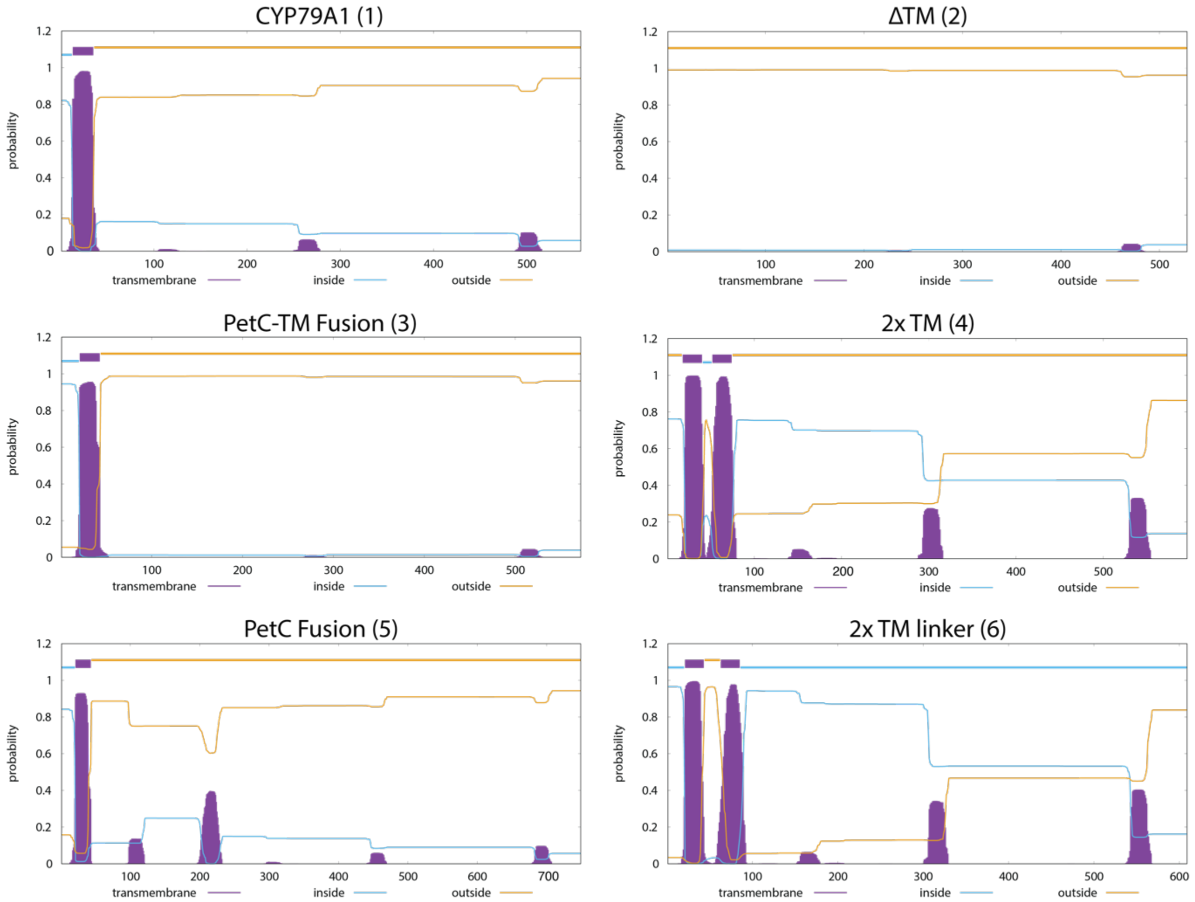

Figure S1. Transmembrane helices and membrane topology prediction by the software THMM 2.0.[1] Predictions for all generated variants of CYP79A1 and fusions thereof with PetC1 (Sll1316) (see Table 1 for details).

(1) [1] Krogh, A.; Larsson, B.; von Heijne, G.; Sonnhammer, E. L. L. Predicting Transmembrane Protein Topology with a Hidden Markov Model: Application to Complete Genomes. *J. Mol. Biol.* **2001**, *305* (3), 567–580.

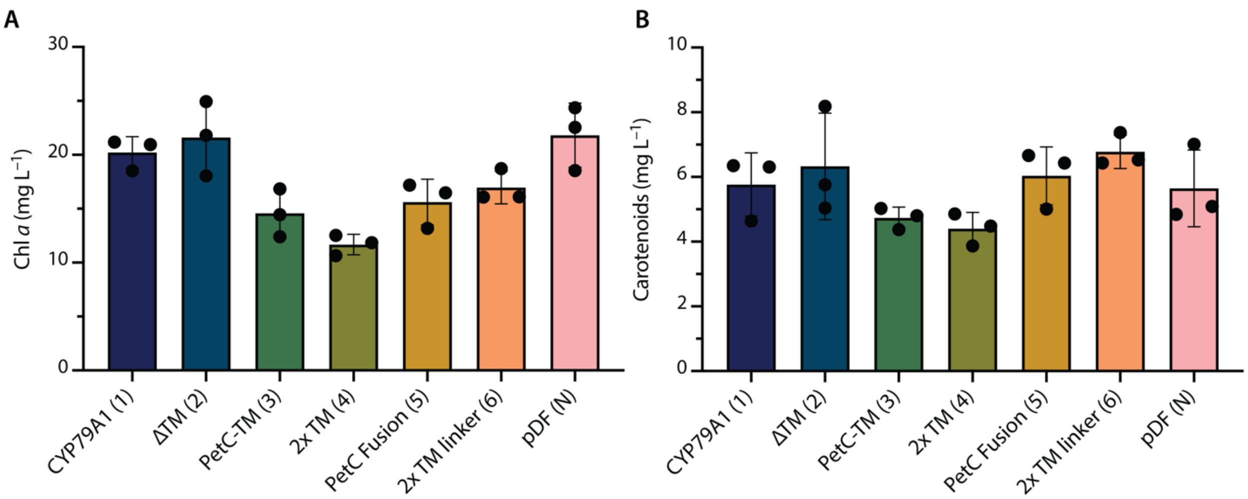

Figure S2. Estimation of pigment content in *Synechocystis* sp. PCC 6803 strains expressing CYP79A1 variants after 7 days of growth. **A** Chlorophyll *a* (Chl *a*) content in mg L^{-1} and **B** carotenoid content in mg L^{-1} are shown. Data shown are averages from n=3 biological replicates, error bars: ±SD.

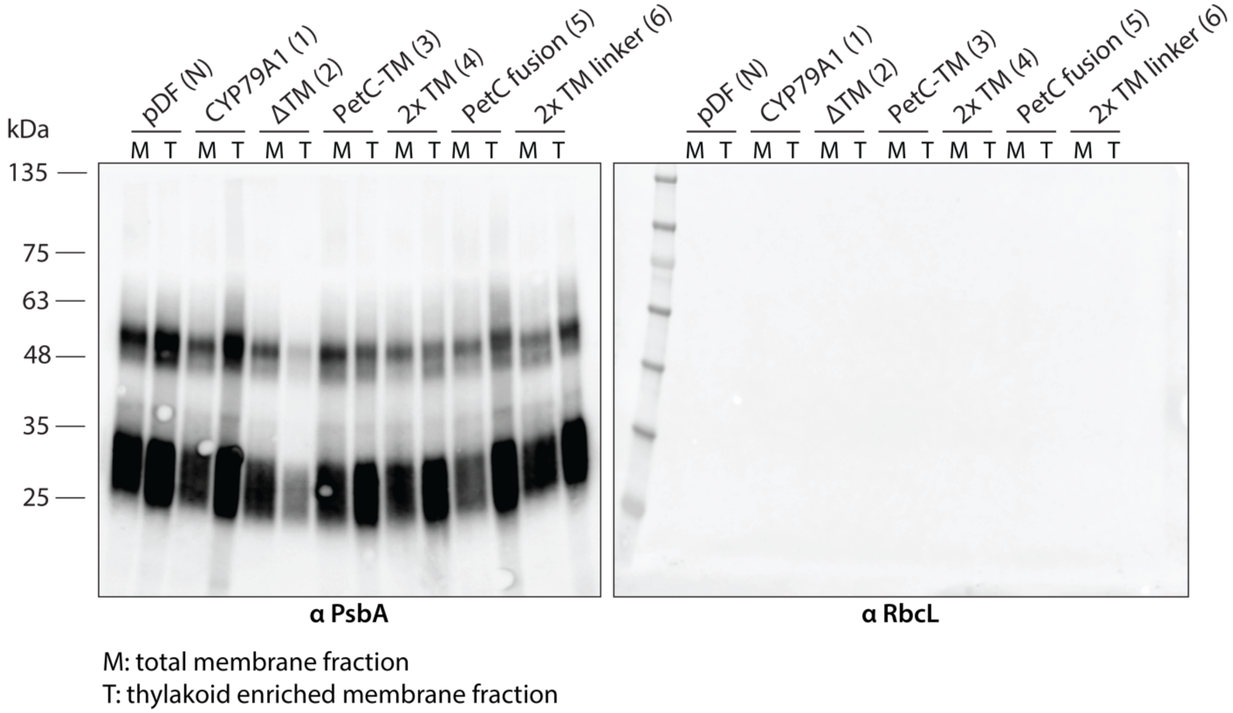

Figure S3. Control blots for membrane fractionation of *Synechocystis* strains expressing CYP79A1 variants. Blots of total membrane (M) and thylakoid enriched membrane fraction (T) for each strain blotted with a PsbA antibody (αPsbA, thylakoid membrane marker) and a RbcL antibody (αRbcL, cytoplasmic marker). 5 µg of estimated protein were loaded for all samples.

Table S1 Primers used for plasmid generation.

Primer	Sequence (5'-3')	Used to generate the following plasmids
PTCYP-1F	CGT CAA GAA TTC ATG GTC ACC TTG T	pJZ94
PTCYP-1R	CAG TCA AAG CTT CTA TTA AAT GGA GAT GGA	
PT-CYP-2F	CGT CAA GAA TTC ATG GCC ACC ATG GAA GTT GA	pJZ95
PT-CYP-2R	CAG TCA AAG CTT CTA TTA AAT GGA GAT GGA GGG ATA CA	
PTCYP-3F	ACA CAG GAA ACA GAC CAT GGA ATT CAT GAC ACA GAT TTC TGG CTC CCC T	pJZ99, pJZ100
PTCYP-3R	CAC GGG CTA AAT AGG ACA AGG TGA CGG GGG GGA TCA AAT ATT TTA CTG CTG GA	pJZ99
PTCYP-4R	GGC GGC TTC AAC TTC CAT GGT GGC TAC TGC TGG ATA GAG GGC C	pJZ100
PTCYP5-ovF	GGC CGG GGC CCT CTA TCC AGC AGT AAA ATA TTT GAT CCC CCC CTC	pJZ101
PTCYP5-ovR	CGG CGG CTT CAA CTT CCA TGG TGG CCC GCG GAT TAT TAT TGT TAT TGT T	
PTCYP6-ovF	GTG GCG GCC GGG GCC CTC TAT CCA GCA GTA GGC GGC GGG AGT GGT GGG GGT GGA AGT GGA GGG GGC AGT	pJZ102
PTCYP6-ovR	GGC GGC GGC GGC TTC AAC TTC CAT GGT GGC ACT GCC CCC TCC ACT TCC ACC CCC ACC ACT CCC GCC GCC	

Table S2. Amino acid sequence of CYP79A1 variants used in this study.

Variant	Amino acid sequence
1<br>(CYP79A1)	MATMEVEAAAATVLAAPLLSSSAILKLLLFVVTLSYLARALRRPRKSTTKCSSTTCASPPAGVGNPPLPPGP<br>VPWPVVGNLPEMLLNKPAFRWIHQMMREMGTDIACVKLGGVHVVSITCPEIAREVLRKQDANFISRPLTFAS<br>ETFSGGYRNAVLSPYGDQWKKMRRVLTSEIICPSRHAWLHDKRTDEADNLTRYVYNLATKAATGDVAVDVRH<br>VARHYCGNVIRRLMFNRRYFGEPQADGGPGPMEVLHMDAVFTSLGLLYAFCVSDYLPWLRGLDLDGHEKIVK<br>EANVAVNRLHDTVIDDRWRQWKSGERQEMEDFLDVLITLKDAQGNPLLTIEEVKAQSQDITFAAVDNPSNAV<br>EWALAEMVNNPEVMAKAMEELDRVVGRERLVQESDIPKLNYVKACIREAFRLHPVAPFNVPHVALADTTIAG<br>YRVPKGSHVILSRTGLGRNPRVWDEPLRFYPDRHLATAASDVALTENDLRFISFSTGRRGCIAASLGTAMSV<br>MLFGRLLQGFTWSKPAGVEAVDLSESKSDTFMATPLVLHAEPRLPAHLYPSISI
2<br>(ΔTM)	MVTLSYLARALRRPRKSTTKCSSTTCASPPAGVGNPPLPPGPVPWPVVGNLPEMLLNKPAFRWIHQMMREMG<br>TDIACVKLGGVHVVSITCPEIAREVLRKQDANFISRPLTFASETFSGGYRNAVLSPYGDQWKKMRRVLTSEI<br>ICPSRHAWLHDKRTDEADNLTRYVYNLATKAATGDVAVDVRHVARHYCGNVIRRLMFNRRYFGEPQADGGPG<br>PMEVLHMDAVFTSLGLLYAFCVSDYLPWLRGLDLDGHEKIVKEANVAVNRLHDTVIDDRWRQWKSGERQEME<br>DFLDVLITLKDAQGNPLLTIEEVKAQSQDITFAAVDNPSNAVEWALAEMVNNPEVMAKAMEELDRVVGRERL<br>VQESDIPKLNYVKACIREAFRLHPVAPFNVPHVALADTTIAGYRVPKGSHVILSRTGLGRNPRVWDEPLRFY<br>PDRHLATAASDVALTENDLRFISFSTGRRGCIAASLGTAMSVMLFGRLLQGFTWSKPAGVEAVDLSESKSDT<br>FMATPLVLHAEPRLPAHLYPSISI
3<br>(PetC-TM)	MTQISGSPDVPDLGRRQFMNLLTFGTITGVAAGALYPAVKYLIPPVTLSYLARALRRPRKSTTKCSSTTCAS<br>PPAGVGNPPLPPGPVPWPVVGNLPEMLLNKPAFRWIHQMMREMGTDIACVKLGGVHVVSITCPEIAREVLRK<br>QDANFISRPLTFASETFSGGYRNAVLSPYGDQWKKMRRVLTSEIICPSRHAWLHDKRTDEADNLTRYVYNLA<br>TKAATGDVAVDVRHVARHYCGNVIRRLMFNRRYFGEPQADGGPGPMEVLHMDAVFTSLGLLYAFCVSDYLPW<br>LRGLDLDGHEKIVKEANVAVNRLHDTVIDDRWRQWKSGERQEMEDFLDVLITLKDAQGNPLLTIEEVKAQSQ<br>DITFAAVDNPSNAVEWALAEMVNNPEVMAKAMEELDRVVGRERLVQESDIPKLNYVKACIREAFRLHPVAPF<br>NVPHVALADTTIAGYRVPKGSHVILSRTGLGRNPRVWDEPLRFYPDRHLATAASDVALTENDLRFISFSTGR<br>RGCIAASLGTAMSVMLFGRLLQGFTWSKPAGVEAVDLSESKSDTFMATPLVLHAEPRLPAHLYPSISI
4<br>(2x TM)	MTQISGSPDVPDLGRRQFMNLLTFGTITGVAAGALYPAVATMEVEAAAATVLAAPLLSSSAILKLLLFVVTL<br>SYLARALRRPRKSTTKCSSTTCASPPAGVGNPPLPPGPVPWPVVGNLPEMLLNKPAFRWIHQMMREMGTDIA<br>CVKLGGVHVVSITCPEIAREVLRKQDANFISRPLTFASETFSGGYRNAVLSPYGDQWKKMRRVLTSEIICPS<br>RHAWLHDKRTDEADNLTRYVYNLATKAATGDVAVDVRHVARHYCGNVIRRLMFNRRYFGEPQADGGPGPMEV<br>LHMDAVFTSLGLLYAFCVSDYLPWLRGLDLDGHEKIVKEANVAVNRLHDTVIDDRWRQWKSGERQEMEDFLD<br>VLITLKDAQGNPLLTIEEVKAQSQDITFAAVDNPSNAVEWALAEMVNNPEVMAKAMEELDRVVGRERLVQES<br>DIPKLNYVKACIREAFRLHPVAPFNVPHVALADTTIAGYRVPKGSHVILSRTGLGRNPRVWDEPLRFYPDRH<br>LATAASDVALTENDLRFISFSTGRRGCIAASLGTAMSVMLFGRLLQGFTWSKPAGVEAVDLSESKSDTFMAT<br>PLVLHAEPRLPAHLYPSISI
5<br>(PetC<br>Fusion)	MTQISGSPDVPDLGRRQFMNLLTFGTITGVAAGALYPAVKYLIPPSSGGSGGGVTAKDALGNDVKVTEFLAS<br>HNAGDRVLAQGLKGDPTYIVVQGDDTIANYGINAVCTHLGCVVPWNASENKFMCPCHGSQYNAEGKVVRGPA<br>PLSLALAHATVTDDDKLVLSTWTETDFRTDEDPWWANNNNNNNNNPRATMEVEAAAATVLAAPLLSSSAIL<br>KLLLFVVTLSYLARALRRPRKSTTKCSSTTCASPPAGVGNPPLPPGPVPWPVVGNLPEMLLNKPAFRWIHQM<br>MREMGTDIACVKLGGVHVVSITCPEIAREVLRKQDANFISRPLTFASETFSGGYRNAVLSPYGDQWKKMRRV<br>LTSEIICPSRHAWLHDKRTDEADNLTRYVYNLATKAATGDVAVDVRHVARHYCGNVIRRLMFNRRYFGEPQA<br>DGGPGPMEVLHMDAVFTSLGLLYAFCVSDYLPWLRGLDLDGHEKIVKEANVAVNRLHDTVIDDRWRQWKSGE<br>RQEMEDFLDVLITLKDAQGNPLLTIEEVKAQSQDITFAAVDNPSNAVEWALAEMVNNPEVMAKAMEELDRVV<br>GRERLVQESDIPKLNYVKACIREAFRLHPVAPFNVPHVALADTTIAGYRVPKGSHVILSRTGLGRNPRVWDE<br>PLRFYPDRHLATAASDVALTENDLRFISFSTGRRGCIAASLGTAMSVMLFGRLLQGFTWSKPAGVEAVDLSE<br>SKSDTFMATPLVLHAEPRLPAHLYPSISI
6<br>(2x TM<br>Linker)	MTQISGSPDVPDLGRRQFMNLLTFGTITGVAAGALYPAVGGGSGGGGSGGGSATMEVEAAAATVLAAPLLSS<br>SAILKLLLFVVTLSYLARALRRPRKSTTKCSSTTCASPPAGVGNPPLPPGPVPWPVVGNLPEMLLNKPAFRW<br>IHQMMREMGTDIACVKLGGVHVVSITCPEIAREVLRKQDANFISRPLTFASETFSGGYRNAVLSPYGDQWKK<br>MRRVLTSEIICPSRHAWLHDKRTDEADNLTRYVYNLATKAATGDVAVDVRHVARHYCGNVIRRLMFNRRYFG<br>EPQADGGPGPMEVLHMDAVFTSLGLLYAFCVSDYLPWLRGLDLDGHEKIVKEANVAVNRLHDTVIDDRWRQW<br>KSGERQEMEDFLDVLITLKDAQGNPLLTIEEVKAQSQDITFAAVDNPSNAVEWALAEMVNNPEVMAKAMEEL<br>DRVVGRERLVQESDIPKLNYVKACIREAFRLHPVAPFNVPHVALADTTIAGYRVPKGSHVILSRTGLGRNPR<br>VWDEPLRFYPDRHLATAASDVALTENDLRFISFSTGRRGCIAASLGTAMSVMLFGRLLQGFTWSKPAGVEAV<br>DLSESKSDTFMATPLVLHAEPRLPAHLYPSISI

Table S3. Densitometry of relative CYP79A1 protein quantities. Estimation of relative CYP79A1 protein quantities (blot shown in Figure 2C) by densitometric analysis using Image Lab Software (Bio-Rad). The band from CYP79A1 (1) was used as a reference band for relative quantitation.

**Strain**	**Lane**	**Protein loaded (µg)**	**Relative quantity**	**Equivalent protein**
CYP79A1 (1)	4	20	1	1
PetC-TM (3)	6	20	1.22	1.22
2x TM (4)	7	20	10.98	10.98
2x TM linker (6)	9	10	10.59	21.18
